# Treatment of Chronic Delta Hepatitis: A Nine-Year Retrospective Analysis

**DOI:** 10.5812/kowsar.1735143X.728

**Published:** 2011-09-01

**Authors:** Serda Gulsun, Recep Tekin, Fatma Bozkurt

**Affiliations:** 1 Department of Infectious Diseases and Clinical Microbiology, Diyarbakir State Hospital, Diyarbakir, Turkey; 2 Department of Infectious Diseases and Clinical Microbiology, Dicle University, Diyarbakir, Turkey

**Keywords:** Chronic delta hepatitis, Pegylated interferon, Treatment

## Abstract

**Background:**

Chronic delta hepatitis is the most severe form of viral hepatitis, for which interferon administration is the only available treatment. However, the efficacy of interferon treatment is affected by the dose and duration of treatment, and relapse rates are high.

**Objectives:**

In this study, we sought to evaluate the efficacy of treatment with pegylated interferon and observe the relapse rates of delta hepatitis after treatment.

**Patients and Methods:**

Forty-six patients with chronic delta hepatitis were retrospectively studied between January 2002 and December 2010. Patients were evaluated for biochemical, virological, and histological responses. They were then followed-up for at least 1 year after discontinuation of the treatment.

**Results:**

All the 46 patients in the study received PEG-IFN therapy. Of the 46 patients,25 were treated with PEG-IFN for 1 year and 21 were treated for 2 years. Sixteen patients(34.7%) showed a biochemical response, 27 (58.6%) showed a virological response, and 39 (84.7%) showed a histological response. Sustained virological and biochemical responseswere achieved in 41% and 47.8% of the patients, respectively. Sixteen (84.2%) patients of the 19 with high levels of hepatitis delta virus RNA (HDV RNA) (HDV RNAlevel > 1 × 105) and 10 (71.4%) of the 14 patients with high titers of hepatitis B surface antigen(HbsAg) (HbsAg > 102 IU/mL) at the beginning of the treatment showed relapse after treatment.

**Conclusions:**

We found no significant differences between 1-year and 2-year treatments.However, the relapse rate was lower in the 2-year treatment group. Higher HDV RNA and HbsAg levels before treatment were associated with higher relapse rates. Younger age was a significant factor in predicting response.

## 1. Background

Chronic delta hepatitis (CDH) is the least common, but the most severe, form of viral hepatitis. It is caused by hepatitis D virus (HDV), which is a defective RNA virus that requires the helper function of hepatitis B virus (HBV) for its assembly and transmission [1][2]. Worldwide,it has been estimated that 15 million HBsAg carriers are infected with HDV. These patients pose a major therapeutic challenge, because most of them have advanced liver disease [2]. CDH may lead to cirrhosis within 2 years in 10–15% of patients and may be frequently associated with hepatocellular carcinoma [3]. Despite recent advances in the treatment of chronic viral hepatitis, therapy of chronic hepatitis D is not yet satisfactory [4]. In particular, the unusual features of HDV, combined with the limited understanding of its unconventional replicative cycle and its high pathogenic potential, make this virus an extremely difficult target for antiviral therapy [2]. Interferon-alpha is the only therapeutic agent currently available for the treatment of chronic hepatitis D and is also the only licensed drug for CDH [4]. However, the rate of relapse is high and the efficacy of treatment is related to the dose and duration of treatment [2][4].

## 2. Objectives

In the current study, we aimed to evaluate the biochemical, virological, and histopatholgical responses of chronic delta hepatitis patients to interferon therapy and to determine the efficacy of pegylated interferon (PEG-IFN) treatment in chronic delta hepatitis. We also monitored the relapse rate of delta hepatitis after treatment.

## 3. Patients and Methods

### 3.1. Patients and Study Settings 

Chronic hepatitis B is endemic, and delta hepatitis is a common infection in the Southeastern part of Turkey. Dicle University and Diyarbakir State Hospital are the 2 major hospitals serving this region. We retrospectively investigated 46 patients with CDH who received treatment and follow-up care in these 2 medical centers from January 2002 to December 2010. All patients underwent a thorough clinical examination and provided detailed medical history. We enrolled patients who were positive for HbsAg and HDV antibodies (IgG-anti-HDV) and positive for serum HDV RNA for at least 6 months, and had chronic hepatitis, as assessed by liver histology. Other inclusion criteria were as follows: age > 18 years; compensated liver function; no previous use of any antiviral therapy; correlation with clinical evaluation; daily alcohol consumption < 50 g; absence of contraindication to antiviral treatment, particularly platelet and polymorphonuclear counts ≥ 75.000/mm3 and 1500/mm3, respectively; absence of coinfections with hepatitis C virus (HCV) or human immunodeficiency virus (HIV); and absence of hepatic decompensation or hepatocellular carcinoma [5]. Informed consent was obtained from all patients who participated in the study. This study was approved by the Ethics Committee of the Dicle University and Administrative Committee of the Diyarbakir State Hospital. The study protocol conforms to the ethical guidelines of the 1975 declaration of Helsinki.

### 3.2. Methods

The diagnosis of CDH was based on the presence of antibodies to the hepatitis delta antigen in the serum and hepatitis delta virus RNA in the serum. All patients were also positive for HbsAg. Serum HDV RNA and HBV DNA levels were determined by a commercial quantitative PCR assay (ABI Prism 7700 Sequence Detection System- real time PCR, USA). Routine serological tests, such as assays for serum alanine (ALT) and aspartate (AST) aminotransferase, direct and total bilirubin, albumin, prothrombin time, complete blood counts, and α-fetoprotein (AFP), were performed by standard methods. Liver ultrasound was performed for all patients. Necroinflammation and fibrosis were assessed with the Knodell histology activity index (HAI) scoring system and the Ishak modification of this system. All biopsy samples with at least 3 portal areas were evaluated to be underscored for necroinflammation and fibrosis. The pathologist was blinded to the patients’ clinical outcomes and biopsy sequence.

### 3.3. Definitions

Alanine aminotransferase normalization and HDV RNA negativity at the end of treatment and at the end of the follow-up period were primary endpoints of the study [5][6]. A viral load reduction greater than 3 logs in the first 3 months of treatment was defined as early virological response (EVR). Sustained virological response (SVR) was defined as undetectable serum HDV RNA 1 year after discontinuation of the last treatment. Relapse was defined as reappearance of serum HDV RNA after treatment. Based on the final virological response, patients were divided into SVR and non-SVR groups. Patients who relapsed after the achievement of the end-of-treatment response were classified as non-SVR. A biochemical response was defined as normal serum ALT levels at the end of treatment or during follow-up. A relapse was defined as an increase in serum ALT levels to more than 1.5 times the upper limit of normal after a biochemical response [5][6]. The endpoints in histologic improvement were; ≥2-point decrease in the Knodell necroinflammation score, and no worsening of the fibrosis score.

### 3.4. Patient Treatment and Follow-up

Pegylated Interferon alpha-2a (PEGASYS, Roche) was administered subcutaneously at a dose of 180 mcg once a week for 48 weeks in 25 patients and for 96 weeks in 21 patients. The injections were reduced or suspended for a while if adverse effects occurred. Outpatients returned for control visits at 2-month intervals. At the control visits, the following assessments were performed; complete physical examination, biochemical tests, tests for HDV RNA, HBV DNA, antibodies to the hepatitis delta antigens, complete blood count, serum AFP and abdominal ultrasonography. The length of the study was calculated from the starting date of antiviral therapy to the last follow-up visit. Liver-related complications (ascites, upper gastrointestinal bleeding, and hepatic encephalopathy) were considered as an endpoint in all patients. Ascites was diagnosed by clinical examination and/or ultrasound detection. Porto-systemic encephalopathy was defined by clinical parameters. The source of gastro-esophageal bleeding was confirmed by endoscopy, whenever possible [7].

### 3.5. Statistical Analysis

Baseline characteristics and measures of clinical and demographic predictors were summarized using the mean, median, standard error, standard deviation (SD), and minimum and maximum values. The results were expressed in terms of hazard ratios with 95% confidence intervals (CI).All analyses were performed using SPSS15.0 program (SPSS 15.0 for Windows Evaluation Version Release 15.0; 06 September 2006).The chi-square test was used in the evaluation of different variables.A P < 0.05 was considered statistically significant.

## 4. Results

Forty-six patients with a mean age of 35.67 ± 11.3 years (range, 20–58 years) were included in our study. Of the 46 patients, 11 (23.9%) were women and 35 (76%) were men. Twelve patients (26%) were HbsAg-positive. Six of the HbeAg-positive patients were in the group that received treatment for 1 year while the rest were in the group that was treated for 2 years. The mean HDV RNA level was 7.05 × 105 (± 3.52 × 105[SD]). All the patients received PEG-IFN treatment and completed the treatment. Three of the patients who were treated with PEG-IFN for 1 year and 2 of the patients who were treated for 2 years completed their treatment by taking half-dose PEG-IFN therapy for certain time periods, because of the adverse effects of the treatment.Biochemical response was observed in 9 patients (36%) in the 1-year treatment group and in 7 patients (33.3%) in the 2-year treatment group. The mean ALT level decreased from 109.16 ± 64.7 to 52.16 ± 30.1 (P < 0.001) in the 1-year treatment group at the end of therapy (EOT), whereas it was decreased from 136.19 ± 91.12 to 73.76 ± 63.8 (P < 0.01) in the 2-year treatment group at the EOT. The mean AST level decreased from 69.8 ± 28.6 to 40.3 ± 21.2 (P = 0.0001) in the 1-year treatment group, while it decreased from 73.9 ± 30.7 to 46.0 ± 38.1 (P = 0.005) in the 2-year treatment group at the EOT.Virological response was observed in 13 patients (52%) in the 1-year treatment group, and in 14 patients (66.6%) in the 2-year treatment group at the EOT. Among patients showing virological responses, 3 patients in the 1-year treatment group and 2 patients in the 2-year treatment group had elevated ALT levels during the follow-up period. The mean HDV RNA level decreased from 2.24 × 105 to 1.4 × 103 (P = 0.008) and from 1.27 × 105 to 3.7 × 103 (P = 0.003) in the 1- and 2-year treatment groups, respectively, at the EOT. At the end of 48 weeks of follow-up, serum HDV RNA was absent in 27 patients (58.6%), and both HBV DNA and HDV RNA were absent in 26 patients (56.5%). Sixteen (84.2%) of the 19 patients with high levels of HDV RNA (HDV RNA level > 1 × 105) and 10 (71.4%) of the 14 patients with high titers of HbsAg (> 102 IU/mL) at the beginning of the treatment showed relapse after treatment (P < 0.0001). Of the 46 patients, 14 (56%) of the 1-year treatment group and 11 (52.3) of the 2-year treatment group had EVR. In EVR patients, the mean ALT level decreased from 133.15 ± 86.4 to 61.41 ± 42.00 (P < 0.0001), and the baseline Knodell score decreased from 11.19 ± 2.209 to 6.65 ± 1.495 (P < 0.0001). Early virological response to PEG-IFN was found to correlate with sustained biochemical response and amelioration of the necroinflammation scores of the liver (P < 0.0001). Sustained virological and biochemical responses were observed in 19 (41%) and 22 (47.8%) patients in the 1- and 2-year treatment groups, respectively. During the follow-up period, of the 19 patients showing SVR, 9 had clinical complaints and 7 had biochemical fluctuations, while of the 27 non-SVR patients (16 in the 1-year and 11 in the 2-year treatment groups, respectively), 25 had clinical complaints and 24 had biochemical fluctuations. We observed more clinical complaints and biochemical fluctuations in non-SVR patients than in SVR patients (P < 0.001).Of the 46 HDV patients, 26 (56.6%) were younger than 35 years, and 20 (43.4%) were older than 35 years. The mean HDV RNA level decreased from 1.33 × 106 to 6.7 × 103 (P < 0.001) in younger patients, while it decreased from 1.4 × 106 to 1.6 × 105 (P = 0.217) in older patients. The baseline Knodell score decreased from 9.85 ± 2.111 to 6.92 ± 2.208 (P < 0.0001) in younger patients and from 10.05 ± 2.282 to 8.2 ± 1.905 (P = 0.01) in older patients. Thus, at the end of treatment, younger patients (< 35 years old) with CDH showed better virological and histological improvement than older CDH patients (P < 0.001).The baseline Knodell score decreased from 9.64 ± 2.325 to 7.0 ± 1.756 (P = 0.0001) in the 1-year treatment group and from 10.29 ± 1.953 to 7.14 ± 2.476 (P = 0.0001) in the 2-year treatment group. No significant difference was observed in histological response rates between the 1-year and 2-year treatment groups. No significant difference was observed in fibrosis scores. Our patients did not develop liver decompensation or HCC. None of the patients lost HbsAg. Seroconversion from HBsAg to anti-HBs was not documented in any of the patients.Seroconversion from HbeAg to anti-HBe was observed in 7 of the 12 patients.

## 5. Discussion

Vaccination against HBV provides protection against HDV, but no effective HDV vaccine has been developed to protect HbsAg-carriers [8]. The course of CDH is generally more severe and patients are generally asymptomatic.Clinically,CDH can manifest as late-stage complications, such as cirrhosis. Alpha-interferon still remains the only available therapeutic agent for treating this disease [8].However, there has been controversy regarding the dose, duration of therapy, and treatment response-rates in CDH. Therefore, the current study is one of the largest studies (n = 46) comparing the efficacy of 1- and 2-year PEG-IFN treatment protocols for CDH patients and examining every aspect of the therapy, including the duration of therapy, biochemical, virological, and histological responses of the patients, and the outcome of the treatment. In Turkey, there are no reports for patients treated for more than 2 years, because the cost of long-term therapy in CDH is not payable by the Government. Hence, these are the groups we compared in our study.Yurdaydin et al. also compared patients receiving 1-year and 2-year treatments in Turkey and reported that 2 years of treatment does not appear to increase sustained response rates in comparison with 1 year of treatment [9]. However, Gunsar et al reported that 2-year treatment is better than 1-year treatment [10]. We investigated 46 CDH patients, a large group for CDH, and observed that there is not much difference between 1-year and 2-year treatments in terms of virological, histological, and biochemical responses. Long-term PEG-IFN therapy might be more efficacious [9][11][12]. Available trials indicate that high doses of interferon and a long duration of therapy were associated with a better biochemical response and improvement in liver histology [11]. To date, it seems that in patients who do respond to interferon therapy, therapy must be extended as long as possible. This seems to be the only treatment choice if the patients can tolerate the adverse effects of pegylated interferon therapy, and monitoring these patients for a sustained response is mandatory.Yurdaydin et al. reported sustained virological and biochemical response rates of 9% and 13%, respectively [9]. Gunsar et al. found virological and biochemical response rates at the end of therapy to be 50% and 60%, respectively [10]. Castelnau et al treated CHD patients with PEG-IFN-alpha 2b, 1.5 μg/kg weekly for 12 months, and reported an SVR of 43% and biochemical response rates of 57% [13]. Niro et al found virological and biochemical response rates at the EOT to be 19% and 37% and sustained virological and biochemical response rates to be 21% and 26%, respectively [14]. However, in 2 other series of patients treated with the same PEG-interferon, an SVR was obtained only in 17% and 21% of the patients [15][16]. The results of the current study appear to show slightly higher virological, biochemical, and histological response rates, at 27 (58.6%), 16 (34.7%), and 39 (84.7%) at the EOT, and sustained virological and biochemical response rates of 41% and 47.8%, respectively. Moreover, we observed more clinical complaints and biochemical fluctuations in non-SVR patients than in SVR patients.We found that younger CDH patients who received the PEG-IFN treatment have a better prognosis, in terms of histological and virological responses, than older CDH patients. We have not seen other reports describing a better prognosis in younger patients. In previous studies,it was reported that patients with a shorter duration of CDH may respond better to therapy, even though clear predictors of response have not yet been identified [2][10]. Moreover, we found a significant correlation between early virological response to PEG-IFN and sustained biochemical response and amelioration of necroinflammation scores of the liver. In recent reports, authors emphasized the importance of EVR in predicting the cases that may need extended treatment[4][13][14][15].One of the striking factors in our study was that higher HDV RNA and HbsAg levels were associated with higher relapse rates in CDH and that the 2-year treatment was not superior to the 1-year treatment.According to our study,an early virological response to PEG-IFN is associated with sustained biochemical response and amelioration in necroinflammation scores of the liver.Younger age was also found to be a significant factor in predicting response.
